# Warm current intensification altered phytoplankton communities in the Yellow Sea: insights from sedimentary ancient DNA metabarcoding

**DOI:** 10.1093/ismeco/ycag172

**Published:** 2026-06-16

**Authors:** Haoyuan Zhang, Yaping Wang, Huiwen Deng, Linxuan Li, Dongyan Liu, Meixun Zhao, Jun Gong

**Affiliations:** School of Marine Sciences, Sun Yat-sen University, and Southern Marine Science and Engineering Guangdong Laboratory (Zhuhai), Zhuhai 519082, China; Guangdong Provincial Key Laboratory of Marine Resources and Coastal Engineering, Zhuhai 519082, China; School of Marine Sciences, Sun Yat-sen University, and Southern Marine Science and Engineering Guangdong Laboratory (Zhuhai), Zhuhai 519082, China; School of Marine Sciences, Sun Yat-sen University, and Southern Marine Science and Engineering Guangdong Laboratory (Zhuhai), Zhuhai 519082, China; School of Marine Sciences, Sun Yat-sen University, and Southern Marine Science and Engineering Guangdong Laboratory (Zhuhai), Zhuhai 519082, China; State Key Laboratory of Estuarine and Coastal Research, Institute of Eco-Chongming, East China Normal University, Shanghai 200241, China; Frontiers Science Center for Deep Ocean Multispheres and Earth System, and Key Laboratory of Marine Chemistry Theory and Technology, Ministry of Education, Ocean University of China, Qingdao 266100, China; Laoshan Laboratory, Qingdao 266237, China; School of Marine Sciences, Sun Yat-sen University, and Southern Marine Science and Engineering Guangdong Laboratory (Zhuhai), Zhuhai 519082, China; Guangdong Provincial Key Laboratory of Marine Resources and Coastal Engineering, Zhuhai 519082, China

**Keywords:** marginal seas, microalgae, paleoecology, Holocene, sequencing, quantitative PCR

## Abstract

Long-term changes in phytoplankton communities are key to understanding and predicting ecosystem responses to climate-driven circulation change. By analyzing sedimentary ancient DNA from two sediment cores in the Yellow Sea, we reconstructed mid-late Holocene phytoplankton dynamics during the intensification of the Yellow Sea Warm Current (YSWC). We found that phytoplankton communities differed significantly between sites, with stronger spatial differentiation in eukaryotes than in cyanobacteria. Dinoflagellates and chlorophytes dominated overall, while Dictyochophyceae (silicoflagellates) were unexpectedly abundant relative to diatoms. Phylogenetic reconstruction recovered diverse Dictyochophyceae lineages (two orders, six genera) dominated by *Pseudopedinella*, indicating greater regional diversity than previously recognized. The cyanobacterial assemblages comprised SIO2C1, UCYN-A, and PCC-6307 lineages. Following the intensification of the YSWC since ~3 ka BP, eukaryotic community structure shifted in the central basin. 18S rRNA gene abundance increased, whereas diversity and silicifier-to-dinoflagellate ratios declined. Although the absolute age of core B07 remains tentative, a pronounced stratigraphic turnover occurred in the frontal zone around 100 cmbsf, with a shift from chlorophyte to dinoflagellate dominance, consistent with reduced freshwater influence and an enhanced YSWC impact. Numerous indicator species exhibited contrasting abundance patterns before and after these transitions. These findings demonstrate the sensitivity of coastal phytoplankton to circulation-driven regime shifts.

## Introduction

The emergence of sedimentary ancient DNA (sedaDNA) technology has revolutionized paleoecological reconstructions by enabling the recovery of genetic material from diverse microbial communities preserved in marine sediments and their responses to environmental changes [1, 2]. Unlike conventional fossil records, sedaDNA captures a broader spectrum of marine microorganisms, including non-fossilizing phytoplankton groups [3, 4]. Despite these advances, most of the investigations using sedaDNA have concentrated on open ocean or polar regions [5–7], its applications in coastal marine environments remain limited [8].

The Yellow Sea is a shallow marginal sea of the western Pacific Ocean. As semi-enclosed basin, it is characterized by complex hydrological conditions, including coastal currents, the Yellow Sea Warm Current (YSWC), and riverine inputs [9]. Following mid-Holocene sea-level stabilization around 7 ka [10], regional syntheses indicate a gradual intensification of the YSWC through the Holocene together with shelf warming and salinification [11]. Quantification of lipid biomarkers in sediment cores from the central South Yellow Sea mud (CYSM) indicates that haptophytes contributed more substantially to total productivity in late Holocene [12], while sea-surface temperature (SST) records displayed an abrupt increase from ~15.6 to ~16.4°C, and the combined biomass of phytoplankton (Diatoms, Dinoflagellates, and Haptophytes) has risen steadily through the Holocene [13]. There was a gradual weakening of the Kuroshio/YSWC system between 6 and 3 ka, followed by a strengthening from 3 to 0 ka [14, 15]. The intensification of the YSWC led to warmer, saltier, overall more oligotrophic but relatively P-enriched and more strongly stratified water masses [16]. Although sedaDNA can reconstruct past phytoplankton dynamics with finer taxonomic resolution than bulk lipid or microfossil indicators, its potential to elucidate how phytoplankton diversity and community structure responded to YSWC variability through the Holocene remains unexplored.

Ocean fronts partition water masses, imposing steep gradients in temperature, salinity, nutrients, and turbulence that re-structure phytoplankton niches: biomass and diversity rise, the diatom–dinoflagellate ratio increases, and community composition shifts toward larger diatoms and fewer prokaryotes [17]. In the Yellow Sea, satellite imagery traces a prominent front east of the Shandong Peninsula where the warm, saline YSWC meets the cooler, fresher, nutrient-rich Yellow Sea Coastal Current (YSCC) [18, 19]. Microfossils in surface sediments mirror this gradient, recording distinct Diatom, Dinoflagellate, and Dictyochophyceae assemblages that confirm spatially structured communities [20]. Yet satellite records span only decades; sediment archives offer centuries to millennia. The long-term response of phytoplankton communities to front migration and YSWC variability remains virtually unexplored.

Here, we present the first sedaDNA reconstruction of phytoplankton communities spanning from the mid-Holocene to the present, based on two cores recovered from the Yellow Sea. By coupling metabarcoding sequencing of 18S and 16S rRNA gene amplicons with quantitative PCR (qPCR) of sedaDNA, we resolve both eukaryotic microalgae and cyanobacteria with unprecedented taxonomic precision. We test two hypotheses: (i) since the mid-Holocene, the phytoplankton assemblages in the central South Yellow Sea mud have responded predominantly to the intensifying YSWC; (ii) along the Shandong Peninsula coast, where coastal currents meet the YSWC, communities reflect the interplay of both water mass, with YSWC influence increasing over time.

## Materials and methods

### Study areas and sampling

**Figure 1:**
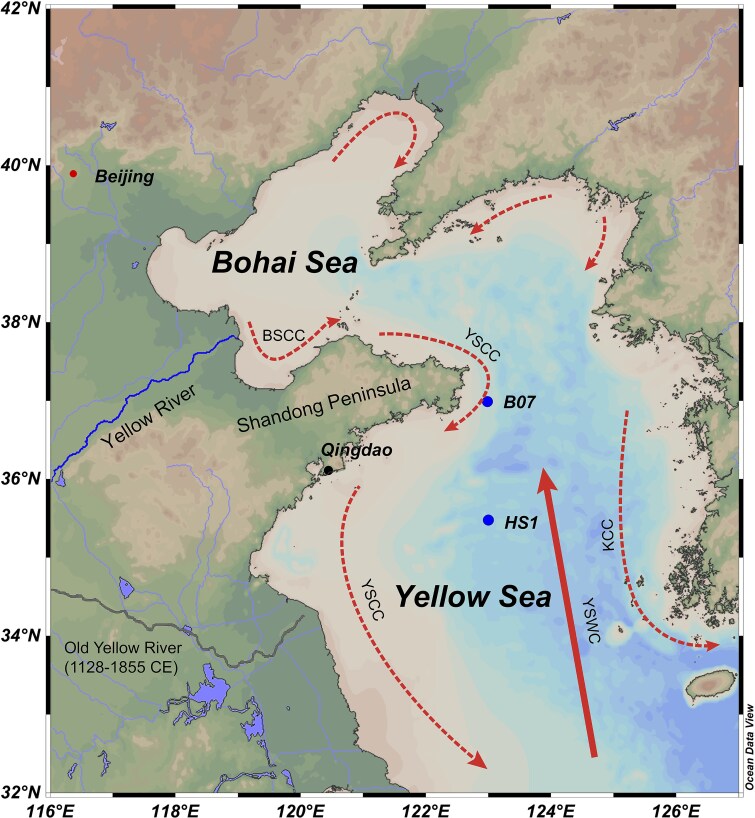
Sampling locations of sediment cores collected in the Yellow Sea. Two dots indicate coring sites: B07 is located along the flow course of the YSCC and near the front of the eastern Shandong Peninsula, while HS1 is situated in the CYSM area, where phytoplankton communities may have been influenced by gradual strengthening of the YSWC. KCC, Korean Coastal Current.

During the NORC 2014-01 cruise aboard the R/V Dongfanghong 2 in May 2014, two sediment cores (HS1 and B07) were collected using a stainless steel gravity core sampler. Core HS1, collected from the muddy area of the central Yellow Sea (123.5°E, 35.5°N), measured 250 cm in length. Core B07, situated off the coast of Chengshan Cape, Shandong Peninsula (123°E, 36.99°N), was 260 cm in length (Fig. 1). The map of sampling was generated using Ocean Data View software [21]. Ocean currents and rivers are adapted from previous studies [9, 22]. The cores were stored at −20°C until transport to the laboratory. Upon arrival, the cores were sliced into 1-cm-thick sections at 10-cm intervals, yielding 25 and 26 subsamples from HS1 and B07, respectively. To minimize contamination, only the inner portion of each core section was subsampled. All subsamples were then stored at −80°C for subsequent sedaDNA extraction and physicochemical analyses.

### Chronological and biogeochemical parameters

For core HS1, the age–depth model for core HS1 was adopted from a companion core collected at the same station during the same cruise (NORC 2014-01). The two HS1 station cores were retrieved in immediate succession at virtually the same position; the companion core was AMS ^14^C-dated on mixed-species benthic foraminifera in a previous study [23], and its published chronology was applied to core HS1 in the present work.

Geochemical parameters, including TOC, TN, δ^13^C, δ^15^N, mean grain size, ammonium (NH_4_^+^), nitrate (NO_3_^−^), nitrite (NO_2_^−^), and metal concentrations (Pb, Cd, As, Mn, Fe, Co, Ni, Cu, Zn, V), were measured following previously described methods [24] with minor modifications (see Supplementary Methods for details). Dissolved inorganic nitrogen (DIN) was calculated as the sum of NH_4_^+^, NO_3_^−^, and NO_2_^−^ concentrations.

### DNA extraction and molecular biological experiments

DNA extraction, PCR amplification, and library preparation were completed within 2 months after sampling. All molecular work was carried out in a dedicated DNA facility following strict contamination-control protocols. Briefly, DNA extraction and PCR setup were performed in a UV-irradiated laminar flow hood using autoclaved consumables and filter-barrier pipette tips, with work surfaces decontaminated by UV-C irradiation and a DNA-degrading reagent before each use. Full details are provided in the Supplementary Methods.

Approximately 0.6 g of each sediment sample was used for DNA extraction. For metabarcoding, the V4-V5 region of eukaryotic 18S rRNA genes was amplified using PCR primers 528F (5′-GCGGTAATTCCAGCTCCAA-3′) and 706R (5′-AATCCRAGAATTTCACCTCT-3′) [25]. The V4 region of bacterial 16S rRNA genes was PCR amplified using primers 515F (5′-GTGCCAGCMGCCGCGGTAA-3′) and 806R (5′-GGACTACHVGGGTWTCTAAT-3′) [26]. Metabarcoding sequencing was performed on an Illumina MiSeq PE300 platform (Novogene, China). The abundances of eukaryotic 18S and bacterial 16S rRNA genes in sedaDNA were determined using qPCR, as previously described [27]. The eukaryotic primers Euk345F (5′-AAGGAAGGCAGCAGGCG-3′) and Euk499R (5′-CACCAGACTTGCCCTCYAAT-3′) [28] and the bacterial primers 341F (5′-CCTACGGGAGGCAGCAG-3′) and 517R (5′-ATTACCGCGGCTGCTGG-3′) [29] were used for qPCR reactions, with no-template controls confirming the absence of contamination. Details of metabarcoding sequencing and qPCR are provided in the Supplementary Methods. Taxon-specific rRNA gene copy numbers were estimated by multiplying the relative reads abundance of each taxon derived from metabarcoding by the total rRNA gene copy number determined by qPCR. This approach has been employed in previous studies [30].

### Sequencing data processing

Raw sequencing data were processed and analyzed using QIIME2 (v.2024.2) [31]. Denoising was performed using the DADA2 algorithm [32]. Taxonomy assignments for eukaryotic and bacterial amplicon sequence variants (ASVs) were conducted against the PR2 (v.5.0.0) [33] and Greengenes2 databases (v.2024.09) [34], respectively. Rarefaction curves were constructed based on the observed number of ASVs against sequencing depth using the “vegan” package in R. For the analysis of phytoplankton groups, eukaryotic data were first verified using AlgaeBase [35] to select pigmented phytoplankton, encompassing both autotrophic and mixotrophic forms, while photosynthetic Cyanobacteria were extracted from the bacterial dataset, with the non-photosynthetic classes Vampirovibrionia and Sericytochromatia removed [36].

Dictyochophyceae (silicoflagellates) sequences were abundant in our datasets, prompting a refined taxonomic classification using phylogenetic analysis. All Dictyochophyceae ASVs were extracted and aligned with closely related reference sequences retrieved from GenBank via BLAST. Maximum likelihood (ML) and Bayesian inference (BI) trees were constructed. As both topologies were nearly identical, a consensus tree was drawn, and ASV placements were validated at a bootstrap value ≥50% or posterior probability ≥0.80. Full details of the alignment, model selection, and tree-building parameters are provided in the Supplementary Methods.

### Statistical analysis

All statistical analyses were conducted in *R* (ver. 4.3.0). ASV richness of phytoplankton were computed using the *vegan* package after rarefaction. Non-metric multidimensional scaling (NMDS) based on Bray–Curtis dissimilarities was used to visualize variations in phytoplankton community structure. Hypothesis testing of the differences in community structure between sample groups were conducted using analysis of similarities (ANOSIM). Nonparametric Mann–Whitney *U* test was applied to assess the significance of differences in physicochemical parameters (e.g. TOC, TN) and biological metrics (e.g. relative abundances, diversity indices) between cores or depth intervals in individual cores. Redundancy analysis (RDA) was used to identify the environmental variables that significantly co-varied with phytoplankton community structure, as previously described [37, 38]. Environmental variables with variance inflation factors (VIF) > 10 were iteratively removed to minimize multicollinearity, and the phytoplankton community matrix was normalized using the Hellinger transformation prior to RDA [39]. To identify a parsimonious subset that significantly explained community variation, forward selection of environmental variables was conducted using the function *ordistep* in *vegan*. The significance of each component (excluding the interaction component) was assessed using 999 permutations [40]. To determine the relative importance of individual variables selected in RDA, hierarchical partitioning (HP) was conducted using the *rdacca.hp* function [41]. Linear regression analysis was performed to examine the relationships between phytoplankton metrics (including diversity indices, relative abundance, and copy numbers) and specific environmental parameters.

## Results

### Chronological data and geochemical parameters

**Figure 2:**
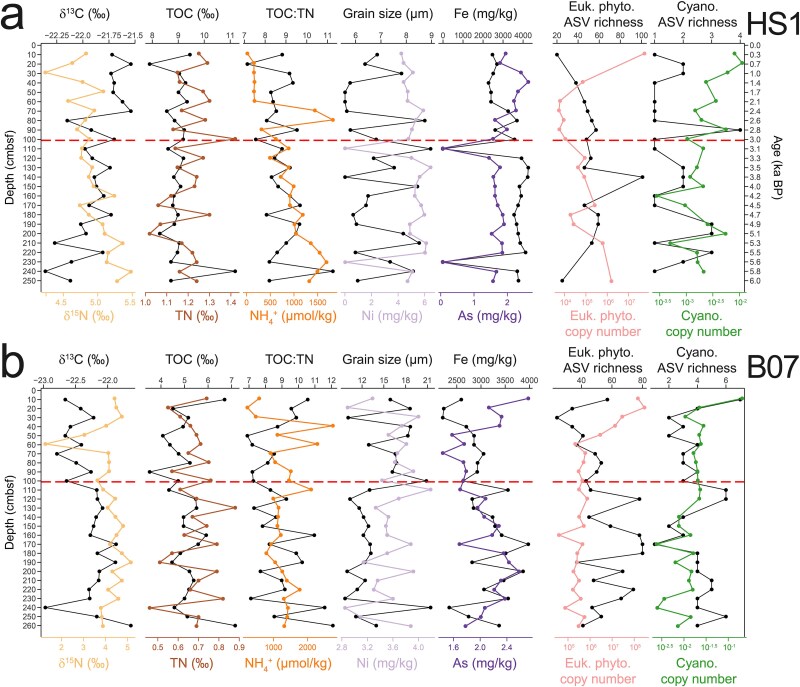
Multiproxy downcore profiles for sediment cores HS1 (a) and B07 (b). Proxies include: stable isotope values (δ^13^C and δ^15^N), total organic carbon contents (TOC), total nitrogen contents (TN), molar TOC:TN ratios, mean grain size, trace metal concentrations (Ni, Fe, and As), 18S rRNA gene abundance and ASV richness of eukaryotic phytoplankton, and 16S rRNA gene abundance and ASV richness of cyanobacteria.

According to the chronological data [23], core HS1 extends from the mid-Holocene (~6.0 ka BP) to the present. The sediment layers in the top of 100 cm below the sea floor (cmbsf) of core HS1 represent ages younger than 3 ka (thereafter referred to as upper segments) (Fig. 2). For core B07, no independent radiometric dating was performed. Instead, a tentative chronological framework was established by applying a surface sediment accumulation rate of 0.16 cm yr^−1^ reported for a nearby site off Chengshan Cape [42]. As a preliminary approximation assuming a constant sedimentation rate, B07 core would span ~1.7 ka, with a depth of 100 cm corresponding to ~0.6 ka (ca. 14th century CE).

Compared with B07, core HS1 contained finer sediments and higher TOC and TN, enriched δ^13^C and δ^15^N, and lower Mn and As concentrations (Fig. 2; Table S1). Within HS1, the upper segments had higher As, but lower concentration of DIN, Fe, Mn, Co, and Zn (in all cases, *P* < .05; Fig. 2a; Table S1). The sediment grain size of core B07 were significantly larger in the upper than in the lower segment (Fig. 2a; Table S1). The upper segments of B07 were also depleted in δ^13^C and showed lower concentrations of Pb, Mn, Fe, and Cu, while V was the only element with higher concentrations (in all cases, *P* < .05; Fig. 2b; Table S1).

### Phytoplankton diversity and community composition across two cores

**Figure 3:**
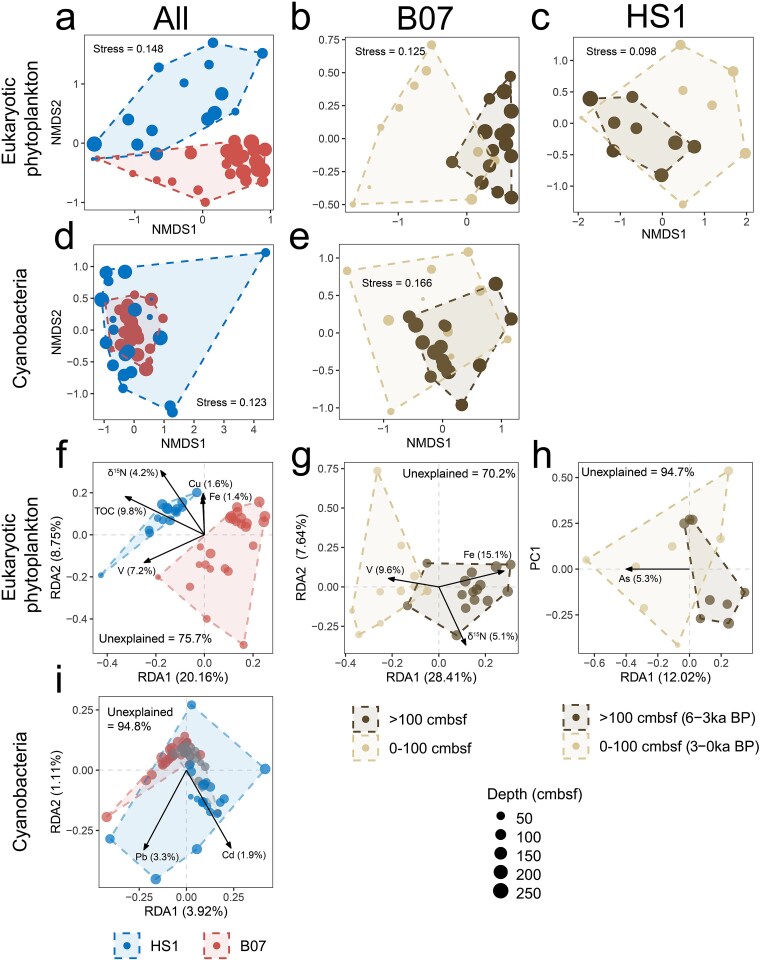
NMDS and RDA analyses of eukaryotic phytoplankton and cyanobacterial communities from sediment cores B07and HS1. NMDS ordinations depict community structure for eukaryotic phytoplankton (a–c) and cyanobacteria (d–e). Panel (a) shows the pooled eukaryotic phytoplankton dataset with two groups corresponding to the two cores (B07 versus HS1); panels (b) and (c) show core-specific NMDS ordinations for B07 and HS1, respectively. Panel (d) shows the pooled cyanobacterial dataset (B07 versus HS1), and panel (e) shows the core-specific NMDS for B07. The cyanobacterial NMDS for HS1 is not shown due to low community complexity. RDA plots identify sedimentary variables significantly associated with community variation for eukaryotic phytoplankton (f–h) and cyanobacteria (i). Panel (f) shows the pooled eukaryotic phytoplankton dataset, whereas panels (g) and (h) show core-specific RDAs for B07 and HS1, respectively; panel (i) shows the RDA for cyanobacteria. No significant explanatory variables were identified for cyanobacteria community structure when each core was analyzed individually. Percentages indicate the proportion of variation explained by each variable.

Across both the eukaryotic and bacterial datasets, the sequencing depth was sufficient (Table S2) and rarefaction curves of all samples reached a clear plateau (Fig. S1). After filtering, 926 133 reads and 1031 ASVs were retained for eukaryotic phytoplankton, and 830 reads and 19 ASVs for cyanobacteria. For cyanobacteria, ASV richness was higher in B07 (3.84 ± 0.29), exceeding HS1 (1.71 ± 0.20) significantly. Eukaryotic phytoplankton 18S rRNA gene copy numbers exhibited distinct depth-related patterns across the two sediment cores, and cyanobacterial 16S rRNA gene copy numbers were generally low, on the order of ~10^–3.5^ to 10^−1^ copies g^−1^ (Fig. 2). NMDS ordinations showed that both eukaryotic phytoplankton and cyanobacterial communities significantly differed between the two sediment cores, which was supported by hypothesis testing using ANOSIM (*P* < .05; Fig. 3a and d; Table S3).

**Figure 4:**
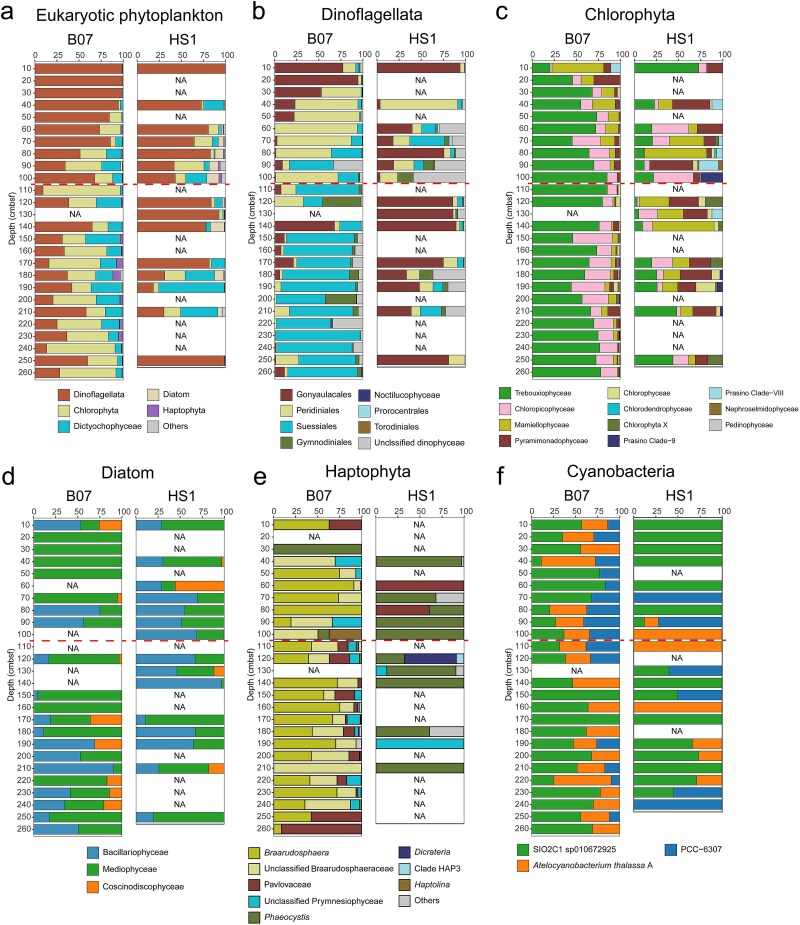
Composition of phytoplankton communities in cores B07 and HS1. (a) Eukaryotic phytoplankton; (b) Dinoflagellata; (c) Chlorophyta; (d) Diatom; (e) Haptophyta; (f) Cyanobacteria. Dashed lines mark the stratigraphic boundaries separating intervals with significantly different community composition. NA, data not available.

Dinoflagellata, Chlorophyta, and Dictyochophyceae dominated eukaryotic phytoplankton communities across both cores (Fig. 4a). Diatoms were consistently minor (typically <5%), and Haptophyta was generally rare (0.5%–1.5%) (Fig. 4a). Other eukaryotic microalgae, Cryptophyta, Chrysophyceae, Chrompodellids, Bolidophyceae, Eustigmatophyceae, Pelagophyceae, Raphidophyceae, and Xanthophyceae were even rarer. Cyanobacterial communities were comprised of SIO2C1 (sp010672925), *Atelocyanobacterium thalassa* A (UCYN-A), and PCC-6307. UCYN-A was more abundant in B07 than in HS1 (*P* = .03; Fig. 4f).

### Classification and occurrence of Dictyochophyceae subgroups buried in the sediments

**Figure 5:**
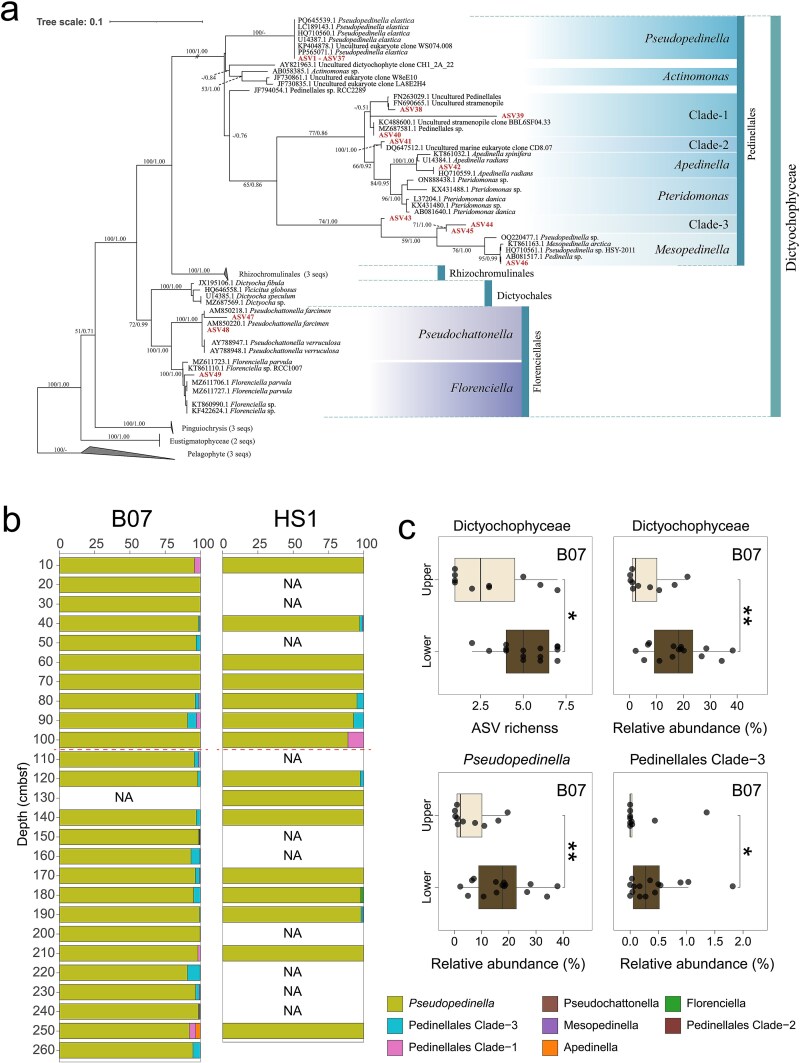
Phylogeny and community patterns of Dictyochophyceae in cores HS1 and B07. (a) Phylogenetic tree of Dictyochophyceae, constructed using Dictyochophyceae ASVs recovered in this study together with publicly available reference sequences. (b) Community composition of Dictyochophyceae in sediment cores HS1 and B07. (c) Boxplots for core B07 showing significant differences in Dictyochophyceae between two groups defined by the 100 cmbsf boundary, based on Dictyochophyceae ASV richness or relative abundance within the eukaryotic phytoplankton community.

A total of 49 ASVs of Dictyochophyceae were extracted for phylogenetic analysis and characterization of distribution (Fig. 5). Thirty-seven ASVs showed high similarities with each other and all clustered with *Pesudopedinella*. Five ASVs (ASV42–ASV46) were placed within order Pedinellales, representing members of genera *Apedinella, Pteridomonas, Mesopedinella*, and three unclassified clades (thereafter named Clade 1 to 3). In addition, two and one ASVs were affiliated with genera *Pseudochattonella* and *Florenciella*, two genera within order Florenciellales (Fig. 5a). *Pseudopedinella elastica* dominated the Dictyochophyceae assemblages. Pedinellales Clade-3 was minor but detected in many sediment layers, whereas others were much rare, presenting only in a few samples (Fig. 5b).

### Changes in phytoplankton community at central Yellow Sea (core HS1)

In HS1, eukaryotic phytoplankton ASV richness increased during 6–3 ka BP and decreased during 3–0 ka BP (Fig. 2a). And we interpret variation in gene abundance as a proxy for eukaryotic phytoplankton biomass preserved in sediments, given that rRNA gene copy numbers of protists scale with cell volume at the single-cell levels [43] and with chlorophyll a concentration at the community levels [30]. In core HS1, eukaryotic phytoplankton rRNA gene abundance declined between 6 and 3 ka, then increased from 3 ka to the present (Fig. 2a), indicating a tipping point in phytoplankton biomass at ~3 ka, consistent with sterol biomarker records [12]. Community structure of eukaryotic phytoplankton differed significantly between pre- and post-3 ka BP intervals (*R* = 0.27, *P* < .05; Fig. 3c; Table S3).

**Figure 6:**
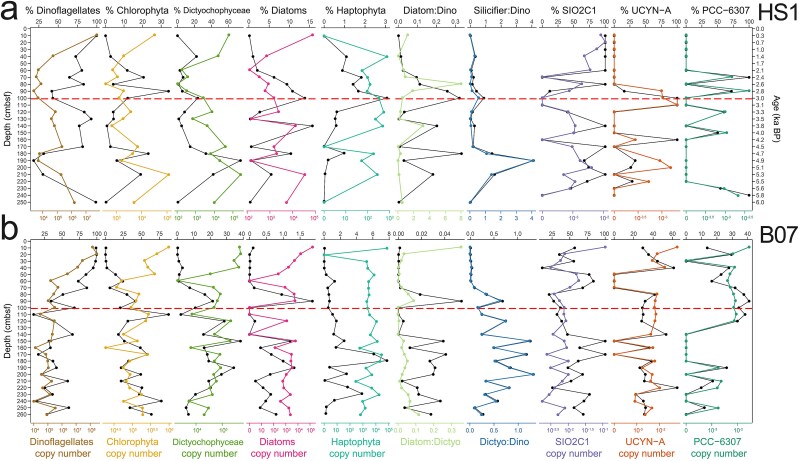
Relative abundances and rRNA genes of major phytoplankton groups and ratios. These include Dinoflagellata, Chlorophyta, Dictyochophyceae, Diatoms, Haptophyta, SIO2C1, UCYN-A, and PCC-6307. The four ratios are Diatoms: Dinoflagellates, Silicifier: Dinoflagellates, Diatoms: Dictyochophyceae, and Dictyochophyceae: Dinoflagellates. (a) Sediment core HS1; (b) core B07. The HS1 panel displays a dual *y*-axes: depth (left) and age (right), adapted from previously published chronologies [23].

**Figure 7:**
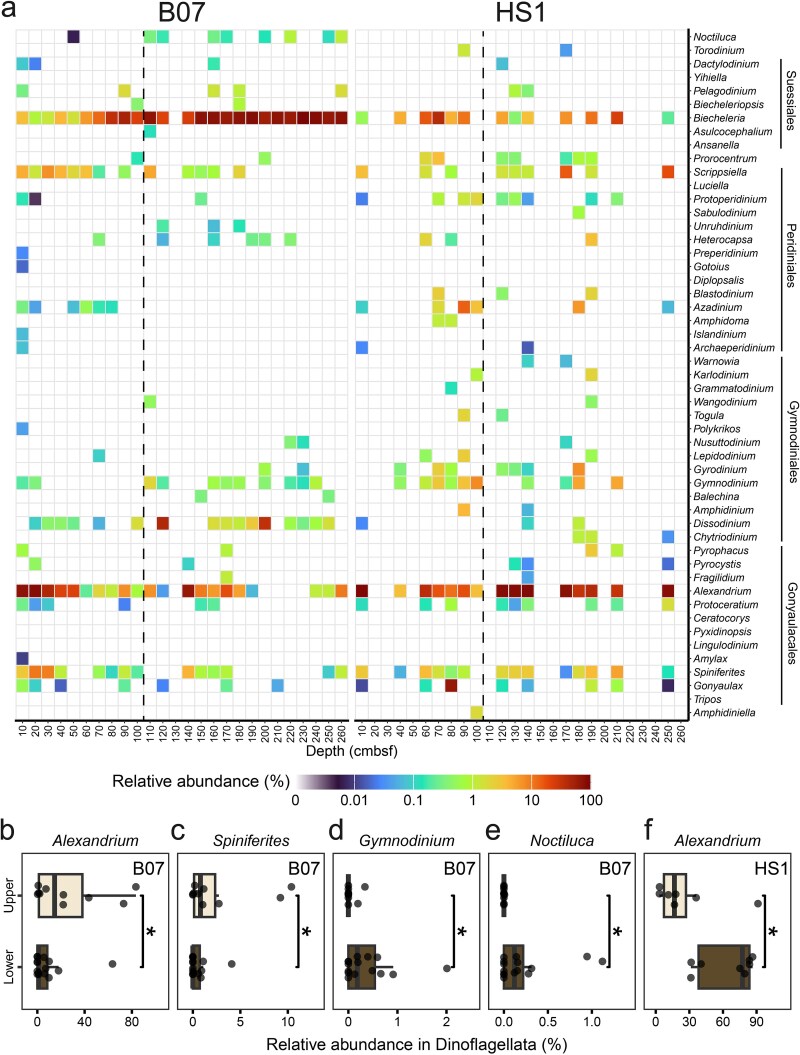
Species dynamics of dinoflagellates in cores B07 and HS1. (a) Heatmap showing the relative abundances of species within dinoflagellate community. (b–e) Boxplots of dinoflagellate species showing significant differences in relative abundance between the upper and lower sections in core B07, with groups defined by the 100 cmbsf boundary. (f) Boxplot of dinoflagellate species showing significant differences in relative abundance between the upper and lower sections in core HS1, with groups defined by the 100 cmbsf (3 ka BP) boundary. Asterisks indicate significance levels (^*^and ^*^^*^indicate *P* < .05 and *P* < .01, respectively).

Dinoflagellates dominated the phytoplankton assemblage in most of core HS1, comprising 69.3% in the upper segment. In the lower segment, although the overall mean relative abundance of dinoflagellates remained high (64.20%), the three deepest samples (210, 190, and 180 cmbsf) were instead dominated by chlorophytes and silicifiers, with dinoflagellate dominance established from 170 cmbsf upward (Fig. 4a; Table S4). Relative abundance of diatoms fluctuated upward during 6–3 ka BP, peaked at ~15% at 3 ka BP, and then decreased during 3–0 ka BP (Fig. 6a). Sequence proportion of Haptophyta was markedly higher in the upper than in the lower sediment segment (*P* < .05; Fig. 4a; Table S4), while Sphaeropleales were absent in the upper sediment segment (0 versus 0.05 ± 0.03% in the lower segment; Table S4). Within the dinoflagellate assemblage as a whole, the relative abundance of *Alexandrium* was significantly lower in the upper segment (*P* < .05; Fig. 7a and f).

The 18S rRNA gene abundance of eukaryotic phytoplankton was relatively low (around 10^4^ copies g^−1^) and stable in the deeper sections of core HS1, with a pronounced increase since ~2 ka and exceeding 10^7^ copies g^−1^ in the uppermost segment (Fig. 2a). The only exceptions included the dinoflagellate order Suessiales and genus *Alexandrium*, which had significantly fewer gene copies in the upper than lower segment (Table S4). Both the Diatom: Dinoflagellata ratio and the silicifiers: Dinoflagellata ratio gradually decreased during 3–0 ka BP (Fig. 6a).

Cyanobacterial ASV richness in core HS1 declined from 1.8 ± 0.2 to 1.6 ± 0.3 upwards (Fig. 2a). The 16S rRNA gene abundance of Cyanobacteria was markedly higher in upper than in lower segment (*P* < .05) (Fig. 2a; Table S4).

### Changes in phytoplankton community at the coast of Yellow Sea (core B07)

Similar to HS1, B07 exhibited increased biomass yet decreased richness in its upper segments compared with lower ones, indicating consistent patterns of phytoplankton change across both cores (Fig. 2b). The historical variations in eukaryotic phytoplankton community seemed to be more pronounced in B07 than those in HS1. Eukaryotic phytoplankton buried in B07 had a significantly lower ASV richness in the upper (42.2 ± 3.2) than in the lower segment (59.9 ± 4.0, *P* < .05; Fig. 2b; Table S4). This decline was principally driven by reduced ASV richness within Chlorophyta, Dictyochophyceae, and Haptophyta (all *P* < .05; Table S4). The community structure of both eukaryotic phytoplankton and cyanobacteria changed significantly across the 100 cmbsf boundary (both *P* < .01; Fig. 3b and e; Table S3).

For relative abundance, dinoflagellates in core B07 was markedly higher in the upper than the lower segment (78.79% versus 34.15%; *P* < .05; Fig. 4a; Table S4), which was mainly attributed by dinoflagellate orders Gonyaulacales and Peridiniales (both *P* < .05; Table S4). In contrast, Suessiales exhibited a lower proportion in the upper segment (*P* < .05; Table S4). Haptophyta was also higher in the lower than the upper segment (*P* < .05; Fig. 4a; Table S4). Marine raphidophyte *Heterosigma akashiwo* was absent in the upper segment (0 versus 0.05 ± 0.01% in the lower segment; Table S4).

The gene abundance of eukaryotic phytoplankton below ~60 cmbsf was nearly 10^5^ copies g^−1^ sediment, and increased continuously upward, reaching ~10^8^ copies g^−1^ at the most surface. Dinoflagellata gene abundance was significantly higher in the upper than the lower segment (*P* < .05; Table S4). In B07, both the Diatom: Dinoflagellata and Dictyochophyceae: Dinoflagellata ratios were significantly lower in the upper than in the lower segments (*P* < .05; Fig. 4; Table S4). The decrease in the Dictyochophyceae: Dinoflagellata ratio was consistent with the trend observed in HS1, although the change in HS1 was not statistically significant (Fig. 4; Table S4).

Within the Dinoflagellate assemblage as a whole, the relative abundance of *Alexandrium* and *Spiniferites* were significantly higher in the upper layer than in the lower layer (*P* < .05; Fig. 7b and c; Table S4), whereas *Gymnodinium* and *Noctiluca* was significantly lower in the upper layer (*P* < .05; Fig. 7d and e; Table S4). For Dictyochophyceae, within the eukaryotic phytoplankton assemblage, the relative abundance of *Pseudopedinella* and Pedinellales Clade 3 were significantly lower in the upper layer than in the lower layer (Fig. 5c).

Relative abundance of PCC-6307, a GTDB-defined cyanobacterial order containing *Synechococcus*-related picocyanobacteria, was much higher in the upper than the lower segment (*P* < .05; Table S4). Cyanobacterial 16S rRNA genes in B07 mostly ranged between ~0.0023 and 0.027 copies g^−1^, with the maximum reaching ~0.21 copies g^−1^ at the 10 cm layer. Like HS1, B07 also exhibited a significantly higher cyanobacterial abundance in the upper (0.039 copies g^−1^) than lower segment (0.011 copies g^−1^; Fig. 2; Table S4).

### Phytoplankton community in association with geochemical properties of sediments

**Figure 8:**
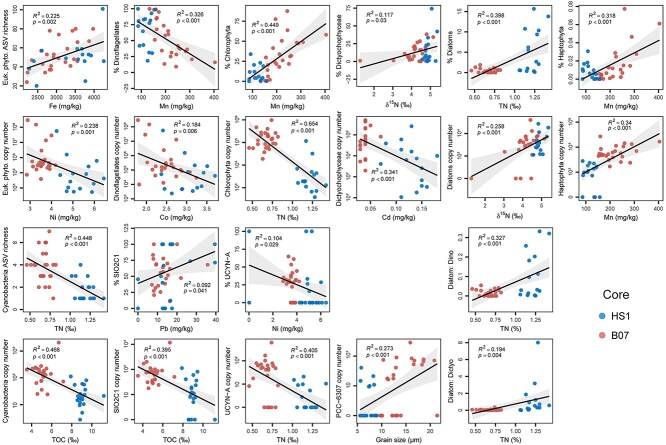
Linear regression analyses between selected environmental variables and phytoplankton community metrics. These include total ASV richness, total rRNA gene abundance, taxon-specific relative abundances, and rRNA gene abundances of major groups. Only correlations significant at *P* < .05 are displayed. The complete results are provided in Table S5.

The geochemical properties of sediments do not necessarily reflect the ambient environment in which phytoplankton grew, but may offer tentative clues to ecological connections—namely, the potential for simultaneous deposition of phytoplankton and certain geochemical constituents. Regression analysis identified the geochemical predictors best explaining community traits of eukaryotic phytoplankton and cyanobacteria (Table S5). Fe, Mn, and Pb explained 22.5%, 11.1%, and 8.3% of the variation in ASV richness of eukaryotic phytoplankton (*P* < .05). Eukaryotic richness was positively correlated with Fe (Fig. 8). In contrast, the variation in cyanobacterial richness was significantly explained by TN (44.8%, negatively related) (Fig. 8, Table S5). TN was identified as the best factor explaining the variations in relative abundance of Diatom (39.8%, positively correlated) in the eukaryotic phytoplankton community (Fig. 8; Table S5). Gene abundance ratios of diatoms to dinoflagellates and of diatoms to Dictyochophyceae were positively related to TN (32.7% and 19.4%, respectively; both *P* < .05; Fig. 8). Interestingly, Mn showed a negative association with the relative abundance of dinoflagellates, but a positive association with Chlorophyta and Haptophyta (Fig. 8). Absolute gene abundances of eukaryotic phytoplankton were significantly explained by two metals Ni and V (Fig. 8; Table S5). TN was the best geochemical variable in explaining the gene abundances of chlorophytes (65.4%) (Fig. 8). δ^15^N positively contributed to the variations in gene abundance of diatoms (25.8%; Fig. 8). The ASV richness and gene abundance of cyanobacteria were explained only by TN (44.8%, negatively) and TOC (45.8%, negatively), respectively (Fig. 8).

RDA and HP showed that the variation in community structure of eukaryotic phytoplankton was mainly driven by TOC, V, δ^15^N, Cu, and Fe (Fig. 3f). For individual cores, As was identified to be a major variable in explaining the variations in HS1 (Fig. 3h). Fe was the key driver of eukaryotic phytoplankton at B07 (Fig. 3g). Cyanobacterial community structure co-varied significantly with Pb and Cd (Fig. 3i).

## Discussion

Here, we present the first sedaDNA-based reconstruction of phytoplankton communities from two sediment cores recovered from the Yellow Sea, spanning the mid-Holocene to the present. Because light is absent in subsurface sediments, phototrophs are unable to grow or reproduce. We therefore interpret the recovered phototroph sequences as deriving from relic DNA, dead cells, and resting cells that were buried in the past. As such, these sequences serve as robust indicators of ancient phytoplankton diversity with minimal contamination from contemporary living populations [1, 2]. Several phytoplankton groups detected in our sedaDNA dataset (such as diatoms, dinoflagellates, and haptophytes) have also been reported from the region through microfossil assemblages and/or sterol-based biomarker records [8, 20, 44]. The overall congruence between sedaDNA, micropaleontological, and geochemical evidence lends confidence to the ecological signals recovered here. Nevertheless, the relative contribution of diatoms and haptophytes in our sedaDNA dataset were lower than those reported from microfossil and sterol records in Yellow Sea sediments [44, 45]. This discrepancy may partly reflect differences in proxy sensitivity and taxonomic coverage. Microfossil counts tend to favor preservable taxa that are relatively large and possess diagnostic features, while lipid biomarkers often provide lower taxonomic resolution. In contrast, sedaDNA is able to detect a broader range of phytoplankton groups, including poorly fossilizing taxa such as Chlorophyta, Cryptophyta, and Cyanobacteria, thereby reducing the relative proportions of diatoms and haptophytes in the overall assemblages.

SedaDNA does not directly capture surface-water activity at any given moment; rather, it integrates biological signals that have been transported through the water column, settled, and variably degraded before burial, and should therefore be read as time-averaged records of communities reaching the seafloor. Post-depositional degradation is a particular concern, as it could bias signals toward younger horizons. In our cores, however, qPCR-quantified eukaryotic 18S rRNA gene copy numbers do not decline monotonically with depth: the deepest horizons retain relatively high copy numbers, whereas the most pronounced decrease occurs within the 6–2 ka interval (Fig. 2a). This pattern is inconsistent with simple exponential decay and instead points to ecologically driven changes in biomass delivery, consistent with a recent Holocene sedaDNA record from the Taiwan Strait based on the same qPCR strategy [8]. We therefore interpret the down-core trends as primarily ecological rather than taphonomic, while acknowledging their integrated nature.

### General characteristics of phytoplankton community in the Yellow Sea

In general, Dinoflagellata and Chlorophyta dominated the eukaryotic phytoplankton communities, consistent with previous metabarcoding surveys in the water and surface sediments in the Yellow Sea [37, 46, 47]. High ASV richness and relative abundance of dinoflagellates were also reported in sedaDNA records from mid–late Holocene cores in the northern South China Sea [8]. This prevalence of dinoflagellates buried in sediment cores may be attribute to their large cell size, high copy numbers of rRNA genes [48], and the ability of many members to form resting cysts that persist in sediments for extended periods [49, 50].

The DNA-based survey identified considerably richer phytoplankton lineages, but also failed to detect certain groups. For instance, only three morphospecies of the Dictyochophyceae genus *Dictyocha* (*Dictyocha fibula, D. messanensis*, and *D. speculum*) were morphologically identified in surface sediments of the Yellow Sea [44, 51], whereas our phylogenetic analysis resolved two orders and six genera of Dictyochophyceae (Fig. 5). The paucity of reported morphospecies partly reflects the limited scope of surface sediments surveys to date. Additionally, many Dictyochophyceae members shed their silica structures prior to encystment [52], complicating morphological identification, while the high sensitivity of PCR may have enabled the detection of trace relic DNA from this group. Together, these observations underscore the strength of sedaDNA in capturing past microbial diversity. Conversely, *Dictyocha* remained undetected in our molecular datasets, likely because eukaryote-universal primers can exhibit mismatches with certain species during PCR amplification [30]. Future work should combine morphological and molecular approaches to resolve such discrepancies.

Both diatoms and Dictyochophyceae are marine silicifiers, with the former usually dominant and the latter much less abundant [53]. A striking finding of our study was that Dictyochophyceae exhibited much higher rDNA sequence proportions than diatoms (averaging 14.4% versus 2.3%) within the buried eukaryotic phytoplankton communities. This contrasts with studies of modern plankton in the Yellow Sea and Bohai Sea, where Dictyochophyceae typically constitute only a very small fraction (∼0.1%) of eukaryotic sequences [47], as well as with global ocean surface waters (<1% relative abundance in Tara Oceans) [53], despite report of high abundances (up to 81%) in sediments from a Japanese coastal lake [54]. Metabarcoding of picoeukaryotes in central Yellow Sea waters showed that the proportion of Dictyochophyceae rDNA sequences (particularly *Pseudochattonella farcimen* and *Florenciella parvula*) increased within the YSWC [55]. These findings suggest that Dictyochophyceae may have been represented a more ecologically important phytoplankton group in the Yellow Sea over the past 3 ka than at present.

### Increased biomass, decreased diversity, and community structure shifts driven by intensified YSWC

Our molecular characterization provides new insights into phytoplankton diversity dynamics during this ecosystem regime shift where diversity was inversely correlated with phytoplankton biomass. This relationship likely reflects changes in hydrography and nutrient regimes. First, intensification of the YSWC introduces warmer waters into the central Yellow Sea, favoring a limited suite of taxa adapted to elevated temperatures while reducing community diversity [56]. Second, the YSWC transports relatively oligotrophic oceanic water onto the continental shelf, weakening shelf-based nutrient supplies and driving the system toward more oligotrophic conditions. Under nutrient-limited regimes, taxa with high nutrient-use efficiency or capabilities to exploit diverse dissolved organic substrates outcompete and exclude species sensitive to nutrient fluctuations or inferior competitors, ultimately reducing community diversity [57]. Third, our multiple linear regression identified Fe as a positive correlate of phytoplankton ASV richness (Fig. 8, Table S5). This relationship is mechanistically sound, as Fe serves as an essential cofactor for key enzymes involved in photosynthesis, respiration, and N₂ fixation; thus, Fe availability strongly regulates phytoplankton growth and physiology [58]. In the upper segments of both HS1 and B07, sedimentary Fe contents declined markedly, likely because the strengthening YSWC reduced riverine and inner-shelf Fe inputs to the central basin, thereby constraining growth in certain phytoplankton groups and reducing overall richness.

Notably, consistent with previously reported centennial-scale phytoplankton community changes from nearby regions [59], both cores record coherent shifts from chlorophyte- or silicifier-dominated to dinoflagellate-dominated communities, indicating that the two sites evolved toward similar ecological states. This long-term transition from chlorophyte- or silicifier-dominated communities to dinoflagellate-dominated communities in the central Yellow Sea appears to have initiated as early as ~4.5 ka (Fig. 4a). SedaDNA evidence of B07 further supports this shift, documenting a transition from chlorophyte to dinoflagellate dominance accompanied by declining silicifier-to-dinoflagellate biomass ratios at the frontal station above 100 cmbsf, suggesting convergence toward the central-basin assemblage. Collectively, these observations reflect intensification of YSWC, which likely promoted oligotrophic conditions favoring dinoflagellates (and other non-diatom groups), analogous to the contrast between diatom-dominated eutrophic Oyashio waters and dinoflagellate/haptophyte-dominated oligotrophic Kuroshio waters in the northwestern Pacific [60].

Our regression analyses indicate that Mn may drive the opposite trends between dinoflagellates and chlorophytes within eukaryotic communities. Mn correlates negatively with dinoflagellate relative abundance, but positively with chlorophyte relative abundance. Mn concentrations decreased significantly in the upper segments in both HS1 and B07. In Yellow Sea sediments, Mn is supplied primarily by terrestrial detrital materials and can be effectively retained in carbonate-associated phases through adsorption onto and incorporation into CaCO_3_, with sedimentary carbonates derived from both Yellow River inputs and biogenic sources [61]. A strengthened YSWC may have altered sediment provenance and composition at our sites, thereby lowering the relative input and/or retention of these Mn-bearing phases and resulting in lower bulk Mn contents. Shifts in Mn availability can alter competitive outcomes among major phytoplankton groups, with dinoflagellates being less sensitive to Mn limitation [62].

Specific phytoplankton groups reflected YSWC intensification differently in each core. In core HS1, the relative abundance of haptophytes (dominated by *Phaeocystis*) increased significantly after 3 ka, consistent with molecular surveys of surface waters showing its widespread distribution in the study area [63], and evidence of increased abundance under warming conditions [64], suggesting a possible link between YSWC-related hydrographic changes and haptophyte expansion. In addition, we observed that increasing influence of open-ocean water masses in the central Yellow Sea was accompanied by a marked increase in Pleurosigmataceae, a known marine diatoms group [65], while Sphaeropleales, a freshwater chlorophyte group [66], gradually disappeared, consistent with a transition toward more marine conditions.

Gene abundances of several dinoflagellate species (*Alexandrium* and *Spiniferites*) declined after ~3 ka in the central Yellow Sea (Fig. 7). *Alexandrium* prefers relatively low temperatures (~10°C) [67], and sediment-trap studies demonstrate that total *Spiniferites* fluxes increase under elevated nutrient availability [68], suggesting that their decline was associated with warming temperatures and increasing nutrient limitation during YSWC intensification.

### Phytoplankton community dynamics at the coastal front

To our knowledge, core B07 provides the first record specifically targeting the long-term variations of marine phytoplankton communities within ocean front. We emphasize that the chronology of core B07 is highly tentative and is intended only to offer readers a first-order temporal reference, rather than a framework for precise age correlation. Because sedimentation rates in coastal settings typically vary through time in response to changes in hydrodynamic conditions, sediment supply, and depositional environments, the assumption of a constant accumulation rate inevitably introduces substantial uncertainties. Consequently, we refrain from making strict chronological comparisons between core B07 and core HS1, and instead focus on the overall trends and community-level shifts recorded in each core.

The sediment grain size of core B07 were significantly larger in the upper than in the lower section (Fig. 2), indicating an intensification of the YSWC in the frontal region, which enhanced current velocities and turbulence, promoting winnowing of fine particles and preferential deposition of coarser material, thereby producing a coarsening-upward trend in the sediments [69]. Our result indicates that the YSWC began to exert a dominant influence along the Shandong coast at around the 100 cmbsf depth. The rRNA gene abundances of both eukaryotic phytoplankton and cyanobacteria were consistently higher in the frontal core (B07) than in the central Yellow Sea core (HS1), suggesting enhanced phytoplankton production and/or deposition at the frontal zone. These abundances were also higher in the upper than in the lower segment of B07 (Fig. 2), which may reflect increase frontal intensity, upwelling, and convergence processes that enhance nutrient supply to surface waters and promote phytoplankton growth [20, 70].

The B07 community shifted from chlorophyte dominance in the lower interval to Dinoflagellate dominance in the upper interval (Fig. 4a). Dinoflagellates likely gain competitive advantages in frontal environments [17], benefiting from rich nutrient supplies via enhanced upwelling. Warming likely contributed to this shift as well, since laboratory experiments indicate that warming (from 22 to 31°C) significantly inhibits photosynthesis and growth of *Picochlorum* [71], a dominant genus exhibiting reduced biomass in the upper segment (Fig. 6a).

Within B07, the dinoflagellate assemblage exhibited a progressive historical transition in dominance from Suessiales to Peridiniales and subsequently to Gonyaulacales (Fig. 4b). Suessiales were rare in HS1 but relatively abundant in B07. Three genera of Suessiales (*Asulcocephalium, Biecheleria*, and *Biecheleriopsis*) have been reported from freshwater samples [72]. The significantly lower relative abundance of these taxa in the upper segment of B07 indicates that phytoplankton community assemblages above 100 cmbsf were increasingly influenced by higher salinity conditions associated with YSWC expansion at the coastal front. *Heterosigma akashiwo* is a low-salinity inhabitant [73], its absence in the upper segment further supports increased salinity driven by intensified YSWC.

At site B07, the relative abundance of PCC 6307 in cyanobacterial communities increased at depth above 100 cmbsf, possibly reflecting an adaptation to elevated salinity [74]. However, cyanobacterial reads were scarce in this study (on average 18 per sample and 830 in total), limiting robust ecological interpretation. Future work should therefore employ taxon-specific primers to achieve sufficient sequencing depth for this ecologically significant group.

## Conclusion

In summary, our sedaDNA records reveal persistent reorganization of Yellow Sea phytoplankton communities since the mid-Holocene. The central South Yellow Sea mud area shows a marked turning point at ~3 ka BP characterized by rising phytoplankton gene abundance and decreasing silicifier-to-dinoflagellate ratios, consistent with a growing imprint of the YSWC and associated shifts in temperature and nutrient supply regimes. The coastal frontal core provides the first millennial-scale evidence that the YSWC began to exert dominant influence along the Shandong coast around the 100 cmbsf, driving pronounced community turnover and convergence toward dinoflagellate-dominated assemblages. These findings demonstrate that frontal regions are not only modern productivity hotspots but also sensitive sedimentary archives capable of capturing long-term reorganization of phytoplankton communities driven by changes in ocean current and water-mass dynamics.

While there is ample evidence to suggest the phytoplankton sedaDNA originates from dead/extracellular DNA and dormant cells (cysts) preserved in sediments, concerns remain regarding potential taxon-specific differences in the degradation of relic DNA versus persistent cysts, which may bias reconstructions of historical phytoplankton diversity. Future studies may apply the recently developed triple metabarcoding approach to distinguish among active, dormant, and dead cells [75, 76], thereby allowing analysis to focus exclusively on cells. Additionally, integrating supplementary genetic markers, morphological and biomarker constraints would enhance our understanding historical changes in coastal ecosystem structure and function.

## Supplementary Material

Supplementary_material_ycag172

## Data Availability

The datasets supporting the conclusions of this article are available in the National Center for Biotechnology Information (NCBI) repository, PRJNA1416884 for cyanobacteria (https://www.ncbi.nlm.nih.gov/bioproject/PRJNA1416884), and PRJNA1416865 for eukaryotic phytoplankton (https://www.ncbi.nlm.nih.gov/bioproject/PRJNA1416865). All data is available for download.
