# Supplementary material for: Warm current intensification altered phytoplankton communities in the Yellow Sea: insights from sedimentary ancient DNA metabarcoding
Source: ISME Commun. 2026 Jun 16;6(1):ycag172. doi: 10.1093/ismeco/ycag172 (PMC13418615; doi:10.1093/ismeco/ycag172)
Supplement: Supplementary_material_ycag172 [file supplementary_material_ycag172.zip › Supplementary Tables.docx]

Table S1. Summary statistics (mean ± SE) and Mann-Whitney U tests comparing core layers in B07 and HS1 for environmental variables. Only significant differences (*p* < 0.05) are highlighted in bold.

| Variables | HS1 | B07 | B07 | | HS1 | |
| --- | --- | --- | --- | --- | --- | --- |
|  |  |  | Upper | Lower | Upper | Lower |
| δ^13^C | **-21.87 ± 0.04** | **-22.30 ± 0.06** | **-22.51 ± 0.06^a^** | **-22.16 ± 0.07^b^** | -21.73 ± 0.06 | -21.97 ± 0.05 |
| δ^15^N | **4.93 ± 0.05** | **4.14 ± 0.14** | 3.81 ± 0.32 | 4.34 ± 0.10 | 4.75 ± 0.07 | 5.05 ± 0.06 |
| TOC | **0.90 ± 0.01** | **0.51 ± 0.01** | 0.48 ± 0.03 | 0.52 ± 0.02 | 0.90 ± 0.01 | 0.90 ± 0.02 |
| TN | **0.12 ± 0.00** | **0.07 ± 0.00^b^** | 0.07 ± 0.00 | 0.07 ± 0.00 | 0.12 ± 0.00 | 0.12 ± 0.00 |
| TOC: TN | 8.78 ± 0.16 | 8.84 ± 0.28 | 8.40 ± 0.41 | 9.11 ± 0.37 | 8.48 ± 0.24 | 8.98 ± 0.21 |
| NH_4_^+^ | **858.1 ± 108.9** | **1258.9 ± 105.2** | 1236.9 ± 250.6 | 1272.5 ± 79.9 | **501.0 ±196.8** | **1096.1 ± 84.7** |
| NO_2_^-^ | 2.89 ± 0.36 | 2.47 ± 0.43 | 2.43 ± 0.52 | 2.50 ± 0.63 | **1.78 ± 0.46** | **3.62 ± 0.44** |
| NO_3_^-^ | **12.26 ± 2.05** | **6.79 ± 1.13** | 7.07 ± 2.29 | 6.61 ± 1.21 | 12.00 ± 3.19 | 12.43 ± 2.75 |
| DIN | **873.23 ± 109.32** | **1268.1 ± 105.4** | 1246.4 ± 251.5 | 1281.7± 79.7 | **514.8 ± 198.9** | **1112.2 ± 83.6** |
| Grain size | **6.84 ± 0.22** | **14.06 ± 0.66** | **16.48 ± 0.98** | **12.55 ± 0.67** | 6.50 ± 0.34 | 7.08 ± 0.29 |
| Pb | **16.19 ± 1.76** | **13.08 ± 1.18** | **9.98 ± 0.92** | **15.02 ± 1.67** | 16.59 ± 0.65 | 15.92 ± 2.94 |
| V | 9.24 ± 0.54 | 9.44 ± 0.16 | **9.87 ± 0.15^a^** | **9.18 ± 0.22** | 10.24 ± 0.28 | 8.57 ± 0.85 |
| Mn | **110.21 ± 8.20** | **216.35 ± 11.86** | **164.15 ± 5.20** | **248.97± 3.65** | **95.17 ± 2.95** | **120.24± 13.1** |
| Fe | 3126.78 ± 227.57 | 3033.96 ± 91.96 | **2698.0 ± 91.4** | **3243.9±110.2** | **2786.9±164.8** | **3353.4±356.6** |
| Co | **2.75 ± 0.18** | **2.44 ± 0.07** | 2.42 ± 0.11 | 2.45 ± 0.09 | **2.69 ± 0.11^a^** | **2.78 ± 0.30** |
| Ni | **4.79 ± 0.31** | **3.55 ± 0.07** | 3.59 ± 0.10 | 3.52 ± 0.09 | 4.86 ± 0.16 | 4.73 ± 0.51 |
| Cu | 6.09 ± 0.37 | 6.22 ± 0.15 | **5.81 ± 0.21** | **6.48 ± 0.18** | 6.61 ± 0.13 | 5.73 ± 0.61 |
| Zn | **17.39 ± 1.20** | **8.30 ± 0.18** | 8.68 ± 0.28 | 8.07 ± 0.23 | **16.23 ± 0.85** | **18.16 ± 1.93** |
| As | **1.71 ± 0.12** | **2.06 ± 0.06** | 1.95 ± 0.13 | 2.13 ± 0.07 | **2.07 ± 0.11** | **1.47 ± 0.15** |
| Cd | **0.13 ± 0.01** | **0.04 ± 0.00** | 0.04 ± 0.00 | 0.04 ± 0.00 | 0.14 ± 0.01 | 0.12 ± 0.01 |

B07: Upper segment (0-100 cmbsf); Lower segment (> 100 cmbsf).

HS1: Upper segment (0-100 cmbsf; 3-0 ka BP); Lower segment (> 100 cmbsf; 6-3 ka BP).

DIN, dissolved inorganic nitrogen; As, arsenic; Cd, cadmium; Co, cobalt; Cr, chromium; Cu, copper; Fe, iron; Mn, manganese; Ni, nickel; Pb, lead; V, vanadium; Zn, zinc.

Units for variables: TN and TOC (molar ratio in %); TOC: TN (molar ratio); grain size (μm); NH_4_^+^, NO_2_^-^, NO_3_^-^ and DIN (μmol kg^-1^ sediment); metals (mg kg^-1^ sediment).

Table S2. Summary of sequence data processing.

|  | Eukaryotic 18S | | | | |  | Bacterial 16S | | | | |
| --- | --- | --- | --- | --- | --- | --- | --- | --- | --- | --- | --- |
| Sample ID | Raw seqs | Filtered seqs | Denoised seqs | Merged seqs | Non-chimeric seqs |  | Raw seqs | Filtered seqs | Denoised seqs | Merged seqs | Non-chimeric seqs |
| B07.010 | 60053 | 58303 | 57396 | 54532 | 52367 |  | 58735 | 57170 | 52875 | 44772 | 44257 |
| B07.020 | 66181 | 64564 | 63933 | 58046 | 54750 |  | 66868 | 65203 | 60924 | 53750 | 53013 |
| B07.030 | 55045 | 53443 | 52828 | 50977 | 47287 |  | 60508 | 58988 | 54542 | 46359 | 45903 |
| B07.040 | 68049 | 65929 | 65487 | 63363 | 62679 |  | 53572 | 52167 | 47993 | 39616 | 39293 |
| B07.050 | 50433 | 48924 | 48646 | 47632 | 45758 |  | 67764 | 66086 | 60729 | 50722 | 50368 |
| B07.060 | 32062 | 30930 | 30753 | 30314 | 29923 |  | 61466 | 59793 | 54793 | 45601 | 45237 |
| B07.070 | 59070 | 57039 | 56445 | 54610 | 54205 |  | 51894 | 50535 | 46455 | 37054 | 36816 |
| B07.080 | 58264 | 56413 | 56143 | 55172 | 54744 |  | 64052 | 62068 | 57288 | 43896 | 43634 |
| B07.090 | 61540 | 59591 | 59053 | 57232 | 57071 |  | 52902 | 51582 | 47100 | 39494 | 39324 |
| B07.100 | 63104 | 60958 | 60657 | 59357 | 59048 |  | 63060 | 61330 | 56714 | 47116 | 46569 |
| B07.110 | 33607 | 32579 | 32338 | 31951 | 30615 |  | 58349 | 56721 | 52228 | 43330 | 42871 |
| B07.120 | 65655 | 63200 | 62483 | 60546 | 56080 |  | 66517 | 64635 | 59379 | 48820 | 48323 |
| B07.140 | 56938 | 55163 | 54674 | 53268 | 52628 |  | 51105 | 49747 | 45727 | 37138 | 36933 |
| B07.150 | 51797 | 50042 | 49714 | 48281 | 47574 |  | 63288 | 61602 | 57360 | 47676 | 47253 |
| B07.160 | 54458 | 52403 | 51725 | 50205 | 49847 |  | 50850 | 49555 | 45306 | 37198 | 37163 |
| B07.170 | 66532 | 63843 | 63252 | 62025 | 60685 |  | 42373 | 41078 | 37067 | 27577 | 27539 |
| B07.180 | 59856 | 57795 | 57179 | 55919 | 54653 |  | 53846 | 52356 | 47764 | 38835 | 38658 |
| B07.190 | 57924 | 55559 | 55057 | 53624 | 53543 |  | 65912 | 64212 | 59475 | 48469 | 48280 |
| B07.200 | 59252 | 57501 | 57050 | 56069 | 54489 |  | 50208 | 48950 | 45310 | 38406 | 38134 |
| B07.210 | 51218 | 49507 | 48924 | 47723 | 47710 |  | 55869 | 54572 | 50476 | 42120 | 41530 |
| B07.220 | 64819 | 62970 | 62451 | 61178 | 58725 |  | 62163 | 60615 | 56610 | 48760 | 48615 |
| B07.230 | 65250 | 63357 | 62755 | 61677 | 60257 |  | 69576 | 67339 | 63183 | 44740 | 44591 |
| B07.240 | 57390 | 55859 | 55342 | 54408 | 53643 |  | 53828 | 52346 | 47442 | 39568 | 39385 |
| B07.250 | 59596 | 57471 | 57164 | 56247 | 56155 |  | 65761 | 63934 | 59064 | 49573 | 49096 |
| B07.260 | 51794 | 50164 | 49717 | 47392 | 47307 |  | 64995 | 63307 | 59105 | 48213 | 48046 |
| HS1.010 | 69440 | 67577 | 66743 | 63419 | 60735 |  | 61972 | 60538 | 55222 | 44228 | 43983 |
| HS1.040 | 69864 | 67427 | 66886 | 65878 | 65653 |  | 45477 | 44241 | 40131 | 31901 | 31819 |
| HS1.060 | 62049 | 60163 | 59577 | 57384 | 57245 |  | 61409 | 59683 | 54930 | 41296 | 41070 |
| HS1.070 | 59247 | 57631 | 57106 | 55697 | 55440 |  | 53155 | 51621 | 47768 | 37781 | 37665 |
| HS1.080 | 68260 | 66318 | 65670 | 63634 | 63281 |  | 64425 | 62808 | 58169 | 48623 | 48440 |
| HS1.090 | 54575 | 52863 | 52467 | 50819 | 49712 |  | 57613 | 56132 | 52221 | 43447 | 43208 |
| HS1.100 | 57178 | 55263 | 55028 | 54122 | 54109 |  | 54572 | 52974 | 47992 | 41638 | 41220 |
| HS1.120 | 67193 | 65298 | 64551 | 61955 | 61128 |  | 60028 | 58389 | 53747 | 45167 | 44929 |
| HS1.130 | 60305 | 58602 | 58099 | 56114 | 55509 |  | 67933 | 66168 | 61765 | 51534 | 50992 |
| HS1.140 | 54848 | 52979 | 52416 | 49762 | 47842 |  | 64821 | 63000 | 58047 | 48808 | 48625 |
| HS1.170 | 65639 | 63593 | 63199 | 61897 | 57102 |  | 54988 | 53423 | 49819 | 41995 | 41714 |
| HS1.180 | 55740 | 53679 | 53206 | 51982 | 51928 |  | 51412 | 49918 | 45894 | 38586 | 38309 |
| HS1.190 | 52609 | 50636 | 50120 | 48960 | 48863 |  | 63875 | 62176 | 56906 | 46651 | 46028 |
| HS1.210 | 56993 | 55253 | 54615 | 53110 | 53020 |  | 69114 | 67401 | 63293 | 54852 | 51158 |
| HS1.250 | 51732 | 50104 | 49861 | 49114 | 48889 |  | 56887 | 55290 | 52150 | 45837 | 45397 |

Table S3. Hypothetical testing of inter-group (between cores) and intra-group (within cores) differences in community structure of both eukaryotic phytoplankton and photosynthetic cyanobacteria using ANOSIM.

|  | Eukaryotic phytoplankton | | Photosynthetic cyanobacteria | |
| --- | --- | --- | --- | --- |
|  | *R* | *p* | *R* | *p* |
| HS1 vs. B07 | 0.47 | < 0.001 | 0.18 | < 0.001 |
| HS1 (0-100 vs. > 100 cmbsf) | 0.27 | 0.031 | -0.01 | 0.511 |
| B07 (0-100 vs. > 100 cmbsf) | 0.56 | < 0.001 | 0.23 | 0.007 |

Table S4. Comparisons of diversity, relative abundance and absolute gene abundances (mean ± SE) of phytoplankton between upper and lower segments of cores B07 and HS1 using Mann-Whitney U tests. Only significant differences (*p* < 0.05) are highlighted in bold.

|  | **B07** | | **HS1** | |
| --- | --- | --- | --- | --- |
|  | Upper | Lower | Upper | Lower |
| **ASV richness** |  |  |  |  |
| Eukaryotic phytoplankton | **42.20±3.23** | **59.93±4.03** | 44.43±4.64 | 55.12±7.59 |
| Dinoflagellata | 21.80±2.93 | 20.07±1.48 | 23.00±2.04 | 26.50±2.24 |
| Chlorophyta | **11.70±1.57** | **22.33±1.61** | 8.71±1.90 | 9.75±1.25 |
| Dictyochophyceae | **3.00±0.71** | **5.20±0.40** | 2.43±0.69 | 3.12±0.90 |
| Diatom | 1.30±0.58 | 2.47±0.41 | 5.71±1.41 | 12.12±6.04 |
| Haptophyta | **1.70±0.42** | **5.33±0.75** | 1.71±0.42 | 1.50±0.50 |
| Cryptophyta | 0.20±0.20 | 0.27±0.12 | 0.14±0.14 | 0.50±0.38 |
| Cyanobacteria | 3.50±0.45 | 4.07±0.37 | 1.56±0.34 | 1.83±0.24 |
| **Ratio** |  |  |  |  |
| Diatom: Dictyochophyceae | 0.06±0.03 | 0.04±0.01 | 1.91±1.04 | 0.59±0.37 |
| Diatom: Dinoflagellata | **0.01±0.01** | **0.02±0.00** | 0.12±0.05 | 0.10±0.04 |
| Dictyochophyceae:  Dinoflagellata | **0.13±0.06** | **0.60±0.10** | 0.17±0.08 | 0.86±0.50 |
| Silicifier: Dinoflagellata | **0.14±0.07** | **0.62±0.10** | 0.28±0.11 | 0.96±0.50 |
| **Relative proportion in eukaryotic phytoplankton (%)** | | | | |
| Dinoflagellata | **78.79±7.03** | **34.15±4.35** | 69.31±8.04 | 64.20±11.35 |
| Gonyaulacales | **26.95±11.04** | **3.99±2.88** | 30.33±13.42 | 49.19±11.59 |
| *Alexandrium* | **24.28±9.90** | **3.71±2.70** | 21.64±12.06 | 47.60±11.50 |
| Suessiales | **7.63±2.19** | **21.91±3.05** | 7.20±3.45 | 2.94±0.93 |
| Peridiniales | **41.59±8.57** | **3.45±1.27** | 16.30±7.98 | 6.53±2.06 |
| Chlorophyta | **13.94±4.59** | **44.46±5.39** | 12.17±4.48 | 7.55±3.04 |
| Trebouxiophyceae | **9.78±3.29** | **31.52±4.76** | 1.98±0.62 | 2.18±1.20 |
| *Picochlorum* sp. | **7.44±2.42** | **19.70±2.92** | 0.54±0.30 | 0.28±0.12 |
| Chloropicophyceae | **2.36±0.88** | **9.26±1.08** | 2.35±0.90 | 0.58±0.20 |
| Chloroparvula B3 sp. | **0.55±0.38** | **1.70±0.38** | 0.00±0.00 | 0.00±0.00 |
| Sphaeropleales | **0.02±0.02** | **0.24±0.06** | **0.00±0.00** | **0.05±0.03** |
| Chlorodendrales X sp. | **0.00±0.00** | **0.02±0.00** | 0.01±0.01 | 0.00±0.00 |
| Dictyochophyceae | **6.36±2.43** | **17.96±2.74** | 9.01±3.77 | 22.54±9.11 |
| *Pseudopedinella* | **6.10±2.27** | **17.45±2.73** | 8.48±3.49 | 22.27±8.97 |
| Diatom | 0.31±0.20 | 0.54±0.11 | 6.19±2.01 | 4.50±2.02 |
| Pleurosigmataceae | 0.00±0.00 | 0.00±0.00 | **1.01±0.35** | **0.03±0.02** |
| Haptophyta | **0.38±0.13** | **2.28±0.58** | **1.42±0.36** | **0.37±0.13** |
| *Braarudosphaera* | **0.24±0.12** | **1.24±0.34** | 0.00±0.00 | 0.00±0.00 |
| Cryptophyta | 0.04±0.04 | 0.02±0.01 | 0.02士0.02 | 0.06士0.04 |
| *Heterosigma akashiwo* | **0.00±0.00** | **0.05±0.01** | 0.00±0.00 | 0.00±0.00 |
| Chrysophyceae Clade EC2H | 0.00±0.00 | 0.01±0.01 | **0.00±0.00** | **0.09±0.04** |
| **Relative abundance in Cyanobacteria (%)** | | | | |
| SIO2C1 | 47.26±7.79 | 60.55±5.62 | 68.00±16.04 | 53.94±11.09 |
| PCC-6307 | **25.47±3.93** | **9.12±3.49** | 19.11±12.85 | 22.05±10.02 |
| *Atelocyanobacterium thalassa*_A | 27.27±6.64 | 30.33±4.22 | 12.89±11.03 | 24.02±10.91 |
| **Eukaryotic phytoplankton 18S rRNA gene abundance [log_10_(x+1) transformed]** | | | | |
| Eukaryotic phytoplankton | **6.56±0.37** | **5.42±0.09** | 4.55±0.55 | 5.07±0.24 |
| Dinoflagellata | **6.44±0.40** | **4.90±0.12** | 4.37±0.59 | 4.81±0.29 |
| Gonyaulacales | **5.35±0.67** | **2.62±0.43** | **3.66±0.70** | **4.61±0.33** |
| *Alexandrium* | **5.30±0.67** | **2.45±0.46** | **3.53±0.71** | **4.58±0.33** |
| Suessiales | 5.23±0.27 | 4.69±0.11 | 2.83±0.61 | 3.09±0.50 |
| Peridiniales | **5.99±0.35** | **3.56±0.21** | 3.58±0.54 | 3.77±0.32 |
| Chlorophyta | 5.25±0.14 | 5.02±0.08 | 3.24±0.23 | 3.64±0.23 |
| Trebouxiophyceae | 5.00±0.12 | 4.85±0.09 | 2.56±0.33 | 2.83±0.34 |
| *Picochlorum* sp. | 4.89±0.13 | 4.65±0.10 | 1.77±0.44 | 1.73±0.42 |
| Chloropicophyceae | 4.34±0.10 | 4.33±0.09 | 2.23±0.34 | 2.60±0.23 |
| Chloroparvula B3 sp. | 2.32±0.52 | 3.46±0.14 | 0.57±0.49 | 0.24±0.24 |
| Sphaeropleales | **0.60±0.40** | **2.32±0.29** | **0.00±0.00** | **0.77±0.31** |
| Chlorodendrales_X_sp. | **0.21±0.21** | **1.04±0.24** | 0.14±0.14 | 0.00±0.00 |
| Dictyochophyceae | 4.94±0.23 | 4.58±0.13 | 3.11±0.40 | 4.05±0.27 |
| *Pseudopedinella* | 4.93±0.23 | 4.56±0.13 | 3.09±0.40 | 4.05±0.27 |
| Diatom | 2.91±0.54 | 2.70±0.30 | 3.03±0.33 | 3.32±0.30 |
| Pleurosigmataceae | 0.00±0.00 | 0.00±0.00 | **1.69±0.29** | **0.50±0.33** |
| Haptophyta | 3.24±0.39 | 3.53±0.16 | 1.89±0.35 | 1.78±0.40 |
| Braarudosphaera | 2.12±0.60 | 3.06±0.28 | 0.00±0.00 | 0.00±0.00 |
| Cryptophyta | 0.33±0.33 | 0.62±0.28 | 0.17±0.17 | 0.54±0.36 |
| *Heterosigma akashiwo* | **0.00±0.00** | **1.34±0.30** | 0.00±0.00 | 0.00±0.00 |
| Chrysophyceae Clade-EC2H | 0.33±0.33 | 0.33±0.23 | **0.00±0.00** | **1.02±0.39** |
| **Cyanobacterial 16S rRNA gene abundance [log_10_(x*10^4^ +1) transformed]** | | | | |
| Cyanobacteria | **2.40±0.11** | **1.96±0.08** | **1.54±0.12** | **1.16±0.10** |
| SIO2C1 | 2.01±0.14 | 1.72±0.06 | 1.20±0.26 | 0.79±0.15 |
| PCC-6307 | **1.68±0.20** | **0.59±0.20** | 0.31±0.21 | 0.36±0.16 |
| *Atelocyanobacterium thalassa*_A | 1.41±0.32 | 1.35±0.16 | 0.22±0.15 | 0.39±0.15 |

B07: Upper segment (0-100 cmbsf); Lower segment (> 100 cmbsf).

HS1: Upper segment (0-100 cmbsf; 3-0 ka BP); Lower segment (> 100 cmbsf; 6-3 ka BP).

Units for variables: Relative abundance (%); Euk. copy number [log_10_(rRNA gene copies g^-1^ sediment +1)]; Cyano. copy number (log_10_(10^-4^ rRNA gene copies g^-1^ sediment +1).

Table S5. Stepwise regressions identify the geochemical variables that best explain the variations in traits of eukaryotic phytoplankton (*n* = 40) and cyanobacteria (*n* = 46) community recorded in the depth layers across two sediment cores.

| Response variable | | Model *R^2^* | Model adjusted *R^2^* | AICc | *P* | Selected predictor | *R^2^* for predictor | Adjusted *R^2^* for predictor | β coefficient  (mean ± SE) | *P* |
| --- | --- | --- | --- | --- | --- | --- | --- | --- | --- | --- |
| Eukaryotic phytoplankton diversity | ASV richness | 0.419 | 0.371 | 210.384 | <0.001 | Fe | 0.225 | 0.205 | 0.009 ± 0.004 | 0.002 |
|  |  |  |  |  |  | Mn | 0.111 | 0.096 | 0.088 ± 0.031 | <0.001 |
|  |  |  |  |  |  | Pb | 0.083 | 0.070 | 0.772 ± 0.341 | <0.001 |
| Ratio of gene abundances | Diatom: Dictyochophyceae | 0.194 | 0.173 | 18.990 | 0.004 | TN | 0.194 | 0.173 | 21.252 ± 7.017 | 0.004 |
|  | Diatom: Dinoflagellates | 0.327 | 0.309 | -208.672 | <0.001 | TN | 0.327 | 0.309 | 1.749 ± 0.408 | <0.001 |
|  | Dictyochophyceae: Dinoflagellates | Not selected |  |  |  |  |  |  |  |  |
|  | Silicifier: Dinoflagellates | Not selected |  |  |  |  |  |  | - |  |
| Relative abundance | %Dinoflagellates | 0.326 | 0.309 | -112.328 | <0.001 | Mn | 0.326 | 0.309 | -0.002 ± 0.001 | <0.001 |
|  | %Chlorophyta | 0.449 | 0.434 | -140.063 | <0.001 | Mn | 0.449 | 0.434 | 0.002 ± 0.000 | <0.001 |
|  | %Dictyochophyceae | 0.117 | 0.094 | -153.109 | 0.030 | δ^15^N | 0.117 | 0.094 | 0.074 ± 0.033 | 0.030 |
|  | %Diatom | 0.531 | 0.491 | -280.143 | <0.001 | TN | 0.398 | 0.382 | 0.631 ± 0.187 | <0.001 |
|  |  |  |  |  |  | Pb | 0.075 | 0.062 | 0.003 ± 0.001 | <0.001 |
|  |  |  |  |  |  | As | 0.058 | 0.047 | -0.034 ± 0.016 | <0.001 |
|  | % Haptophyta | 0.413 | 0.382 | -343.615 | <0.001 | Mn | 0.318 | 0.300 | 0.000 ± 0.000 | <0.001 |
|  |  |  |  |  |  | Cd | 0.096 | 0.082 | 0.137 ± 0.056 | <0.001 |
| Absolute abundance  (rRNA gene copy number per gram sediment) | Lg (Euk. Phytoplankton+1) | 0.437 | 0.407 | -8.963 | <0.001 | Ni | 0.238 | 0.217 | -0.795 ± 0.157 | <0.001 |
|  |  |  |  |  |  | V | 0.199 | 0.190 | 0.689 ± 0.190 | <0.001 |
|  | Lg (Dinoflagellates+1) | 0.512 | 0.472 | -4.866 | <0.001 | Co | 0.184 | 0.163 | -1.336 ± 0.369 | 0.006 |
|  |  |  |  |  |  | V | 0.239 | 0.229 | 0.903 ± 0.201 | <0.001 |
|  |  |  |  |  |  | Grain size | 0.089 | 0.080 | 0.091 ± 0.036 | <0.001 |
|  | Lg (Chlorophyta+1) | 0.706 | 0.690 | -47.947 | <0.001 | TN | 0.654 | 0.645 | -30.616 ± 3.275 | <0.001 |
|  |  |  |  |  |  | V | 0.052 | 0.045 | 0.299 ± 0.117 | <0.001 |
|  | Lg (Dictyochophyceae+1) | 0.465 | 0.436 | -24.942 | <0.001 | Cd | 0.341 | 0.323 | -15.737 ± 2.777 | <0.001 |
|  |  |  |  |  |  | δ^15^N | 0.124 | 0.113 | 0.550 ± 0.188 | <0.001 |
|  | Lg (Diatoms+1) | 0.258 | 0.239 | 6.459 | <0.001 | δ^15^N | 0.258 | 0.239 | 0.876 ± 0.241 | <0.001 |
|  | Lg (Haptophyta+1) | 0.416 | 0.384 | -1.102 | <0.001 | Mn | 0.340 | 0.323 | 0.006 ± 0.003 | <0.001 |
|  |  |  |  |  |  | TOC | 0.075 | 0.061 | -2.080 ± 0.953 | <0.001 |
| Cyanobacteria diversity | ASV richness | 0.448 | 0.435 | 19.724 | <0.001 | TN | 0.448 | 0.435 | -38.977 ± 6.527 | <0.001 |
| Relative abundance | %SIO2C1 | 0.092 | 0.071 | 320.421 | 0.041 | Pb | 0.092 | 0.071 | 1.260 ± 0.598 | 0.041 |
|  | %Atelocyanobacterium_thalassa_A | 0.104 | 0.083 | 302.782 | 0.029 | Ni | 0.104 | 0.083 | -6.880 ± 3.052 | 0.029 |
|  | %PCC_6307 | Not selected |  |  |  |  |  |  |  |  |
| Absolute abundance  (*10^-4^ rRNA gene copy number per gram sediment) | Lg (cyanobacteria+1) | 0.468 | 0.456 | -79.239 | <0.001 | TOC | 0.468 | 0.456 | -1.818 ± 0.292 | <0.001 |
|  | Lg (SIO2C1+1) | 0.649 | 0.624 | -77.484 | <0.001 | TOC | 0.395 | 0.381 | -2.007 ± 0.374 | <0.001 |
|  |  |  |  |  |  | V | 0.085 | 0.075 | 0.096 ± 0.036 | 0.011 |
|  |  |  |  |  |  | Ni | 0.147 | 0.145 | -0.445 ± 0.109 | <0.001 |
|  |  |  |  |  |  | δ^15^N | 0.022 | 0.023 | -0.217 ± 0.101 | 0.037 |
|  | Lg (Atelocyanobacterium_thalassa_A +1) | 0.405 | 0.391 | -36.051 | <0.001 | TN | 0.405 | 0.391 | -19.475 ± 3.560 | <0.001 |
|  | Lg (PCC_6307+1) | 0.273 | 0.257 | -28.903 | <0.001 | Grain size | 0.273 | 0.257 | 0.097 ± 0.024 | <0.001 |
