# Supplementary material for: Warm current intensification altered phytoplankton communities in the Yellow Sea: insights from sedimentary ancient DNA metabarcoding
Source: ISME Commun. 2026 Jun 16;6(1):ycag172. doi: 10.1093/ismeco/ycag172 (PMC13418615; doi:10.1093/ismeco/ycag172)
Supplement: Supplementary_material_ycag172 [file supplementary_material_ycag172.zip › Supplementary Methods.docx]

Supplementary methods

1 Biogeochemical parameters

Total organic carbon (TOC) and total nitrogen (TN) contents were determined using a Vario Micro Cube elemental analyzer (Elementar, Germany). Stable isotopes δ^13^C and δ^15^N were analyzed using a mass spectrometer (MAT253, Thermo Fisher Scientific, USA). Sediment mean grain size was determined using a Malvern Mastersizer 2000F granulometer (Malvern, England). Ammonium (NH_4_^+^), nitrate (NO_3_^−^), and nitrite (NO_2_^−^) were extracted from lyophilized sediments using a 2M KCl solution at a sediment-to-extractant ratio of 1:10 and analyzed using a nutrient AutoAnalyzer (Seal, Germany). After pre-treatment with 1 M HCl, metal concentrations (Pb, Cd, As, Mn, Fe, Co, Ni, Cu, Zn, V) of sediments were determined using an ELAN DRC II plasma mass spectrometer (PerkinElmer, Hong Kong).

2 DNA extraction and molecular biological experiments

All molecular biological procedures were performed in a dedicated molecular biology laboratory. DNA extraction and PCR setup were conducted in a UV-equipped laminar flow hood. Prior to each use, the hood and work surfaces were irradiated with UV-C light for 30 min and wiped down with a DNA-degrading reagent to eliminate residual nucleic acids. All consumables and non-disposable tools were autoclaved (121 ℃, 20 min) before use. Filter-barrier pipette tips were used throughout to prevent aerosol contamination.

Approximately 0.6 g of each sediment sample was used for DNA extraction. The detailed protocols for DNA extraction and metabarcoding sequencing followed [1]. In brief, sediment DNA was extracted and purified using the FastDNA Spin Kit (MP Biomedical, USA) according to the manufacturer’s instructions. For metabarcoding, the V4-V5 region of eukaryotic 18S rRNA genes was amplified using PCR primers 528F (5′-GCGGTAATTCCAGCTCCAA-3′) and 706R (5′-AATCCRAGAATTTCACCTCT-3′) [2]. The V4 region of bacterial 16S rRNA genes was PCR amplified using primers 515F (5′-GTGCCAGCMGCCGCGGTAA-3′) and 806R (5′-GGACTACHVGGGTWTCTAAT-3′) [3]. To facilitate sample multiplexing, 6-bp barcodes were attached to the forward primers. PCR was conducted with an initial denaturation at 98 °C for 1 min; 30 cycles of 98 °C for 10 s, 50 °C for 30 s, and 72 °C for 30 s; followed by a final extension at 72 °C for 5 min. PCR products were purified with a GeneJET Gel Extraction Kit (Thermo Scientific), and sequencing libraries were generated using the Illumina TruSeq DNA PCR-Free Library Preparation Kit (Illumina, USA). Sequencing was performed on an Illumina MiSeq PE300 platform (Novogene, China).

The abundances of eukaryotic 18S and bacterial 16S rRNA genes in sedaDNA were determined using qPCR, as previously described [4]. The eukaryotic primers Euk345F (5′-AAGGAAGGCAGCAGGCG-3′) and Euk499R (5′-CACCAGACTTGCCCTCYAAT-3′) [5] and the bacterial primers 341F (5′-CCTACGGGAGGCAGCAG-3′) and 517R (5′-ATTACCGCGGCTGCTGG-3′) [6], and SYBR Premix Ex TaqTM II (TaKaRa, Dalian) were used for qPCR reactions. The reaction system (20 μl) included 0.4 μl forward primer (final concentration: 0.1 μM), 0.4 μl reverse primer (final concentration: 0.1 μM), 0.4 μl RoxDyeⅡ, 1 μl DNA template, 8.5 μl ddH_2_O and 10 μl SYBR. The reactions were performed in a real-time fluorescence quantitative PCR instrument (ABI 7500, USA). For eukaryotic 18S rRNA genes, qPCR was performed with an initial denaturation at 95 °C for 7 min, followed by 40 cycles of 95 °C for 15 s, 60 °C for 60 s, and 77 °C for 25 s, and a final dissociation stage of 95 °C for 15 s, 60 °C for 60 s, 97 °C for 1 s, and 37 °C for 30 s. For bacterial 16S rRNA genes, the program consisted of an initial denaturation at 95 °C for 7 min, followed by 40 cycles of 95 °C for 30 s, 57 °C for 30 s, and 72 °C for 30 s, and a dissociation stage of 95 °C for 15 s, 60 °C for 60 s, 95 °C for 1 s, and 37 °C for 30 s.Each sample was analyzed in triplicate, and the average value was used for quantification. No-template controls (NTCs), in which nuclease-free water replaced the DNA template, were included on every qPCR plate. All NTCs consistently yielded no detectable amplification signal, confirming the absence of exogenous DNA contamination in the reaction system.

To approximate the absolute abundance of individual taxa, taxon-specific rRNA gene copy numbers were estimated by multiplying the relative reads abundance of each taxon derived from metabarcoding by the total rRNA gene copy number of the corresponding sample determined by qPCR. This approach has been employed in previous environmental DNA studies [7].

**3 Phylogenetic analysis**

Dictyochophyceae, commonly known as silicoflagellates, are silica-secreting protists. Their sequences turned out to be abundant in our datasets, which promoted us to refine their taxonomic classification using phylogenetic analysis. Briefly, all amplicon sequence variants (ASVs) of dictyochophyceae were extracted and queried against GenBank using BLAST, to retrieve the closely related, relatively long 18S rRNA gene sequences, which severed reference sequences and analyzed together with the dictyochophyceae ASVs. All the sequences were aligned using ClustalW with default parameters [8] and manually refined using BioEdit v7.2.5 [9], yielding a final alignment of 1,620 nucleotide positions. Maximum likelihood (ML) analyses were run under the GTR+I+G model, with RAxML-HPC [10] on the CIPRES Science Gateway [11], using 1,000 nonparametric bootstrap replicates to assess nodal support. Bayesian inference (BI) was conducted with MrBayes v3.2.7 [12] on CIPRES, with model selection informed by MrModeltest [13]. Two independent MCMC runs, each comprising four chains, were executed for 1,000,000 generations, sampling every 100 generations; the first 25% of samples were discarded as burn-in, and convergence between runs was confirmed prior to summarizing the posterior distribution. Resulting ML and BI topologies were visualized in iTOL [14]. As both trees displayed nearly identical topologies, a consensus tree was drawn. The placements of dictyochophyceae ASVs were validated at a bootstrap value ≥ 50% or posterior probability ≥ 0.80.

**References**

1. Li G et al. Quantifying relative contributions of biotic interactions to bacterial diversity and community assembly by using community characteristics of microbial eukaryotes. *Ecol Indic* 2023;**146**:109841. https://doi.org/10.1016/j.ecolind.2022.109841

2. Cheung MK et al. Composition and genetic diversity of picoeukaryotes in subtropical coastal waters as revealed by 454 pyrosequencing. *ISME J* 2010;**4**:1053–1059. https://doi.org/10.1038/ismej.2010.26

3. Caporaso JG et al. Global patterns of 16S rRNA diversity at a depth of millions of sequences per sample. *Proc Natl Acad Sci U S A* 2011;**108**:4516–4522. https://doi.org/10.1073/pnas.1000080107

4. Sun F et al. Seagrass (Zostera marina) colonization promotes the accumulation of diazotrophic bacteria and alters the relative abundances of specific bacterial lineages involved in benthic carbon and sulfur cycling. *Appl Environ Microbiol* 2015;**81**:6901–6914. https://doi.org/10.1128/AEM.01382-15

5. Zhu F et al. Mapping of picoeucaryotes in marine ecosystems with quantitative PCR of the 18S rRNA gene. *FEMS Microbiol Ecol* 2005;**52**:79–92. https://doi.org/10.1016/j.femsec.2004.10.006

6. Muyzer G, de Waal EC, Uitterlinden AG. Profiling of complex microbial populations by denaturing gradient gel electrophoresis analysis of polymerase chain reaction-amplified genes coding for 16S rRNA. *Appl Environ Microbiol* 1993;**59**:695–700.

7. Xu S et al. Diversity, community structure, and quantity of eukaryotic phytoplankton revealed using 18S rRNA and plastid 16S rRNA genes and pigment markers: a case study of the Pearl River Estuary. *Mar Life Sci Tech* 2023;**5**:415–430. https://doi.org/10.1007/s42995-023-00186-x

8. Thompson JD, Higgins DG, Gibson TJ. CLUSTAL W: improving the sensitivity of progressive multiple sequence alignment through sequence weighting, position-specific gap penalties and weight matrix choice. *Nucleic Acids Res* 1994;**22**:4673–4680. https://doi.org/10.1093/nar/22.22.4673

9. Hall TA. BioEdit: a user-friendly biological sequence alignment editor and analysis program for Windows 95/98/NT. *Nucleic acids symp ser* 1999;**41**:95–98.

10. Stamatakis A. RAxML version 8: a tool for phylogenetic analysis and post-analysis of large phylogenies. *Bioinformatics* 2014;**30**:1312–1313. https://doi.org/10.1093/bioinformatics/btu033

11. Miller MA, Pfeiffer W, Schwartz T. Creating the CIPRES Science Gateway for inference of large phylogenetic trees. *2010 Gateway Computing Environments Workshop (GCE)*. 2010. 2010, pp 1–8.

12. Ronquist F et al. MrBayes 3.2: efficient bayesian phylogenetic inference and model choice across a large model space. *Syst Biol* 2012;**61**:539–542. https://doi.org/10.1093/sysbio/sys029

13. Nylander J. MrModeltest v2. 2004. Uppsala: Evolutionary Biology Centre, Uppsala University, 2004.

14. Letunic I, Bork P. Interactive Tree of Life (iTOL) v6: recent updates to the phylogenetic tree display and annotation tool. *Nucleic Acids Res* 2024;**52**:W78–W82. https://doi.org/10.1093/nar/gkae268
