# Supplementary figures and images for: Warm current intensification altered phytoplankton communities in the Yellow Sea: insights from sedimentary ancient DNA metabarcoding
Source: ISME Commun. 2026 Jun 16;6(1):ycag172. doi: 10.1093/ismeco/ycag172 (PMC13418615; doi:10.1093/ismeco/ycag172)

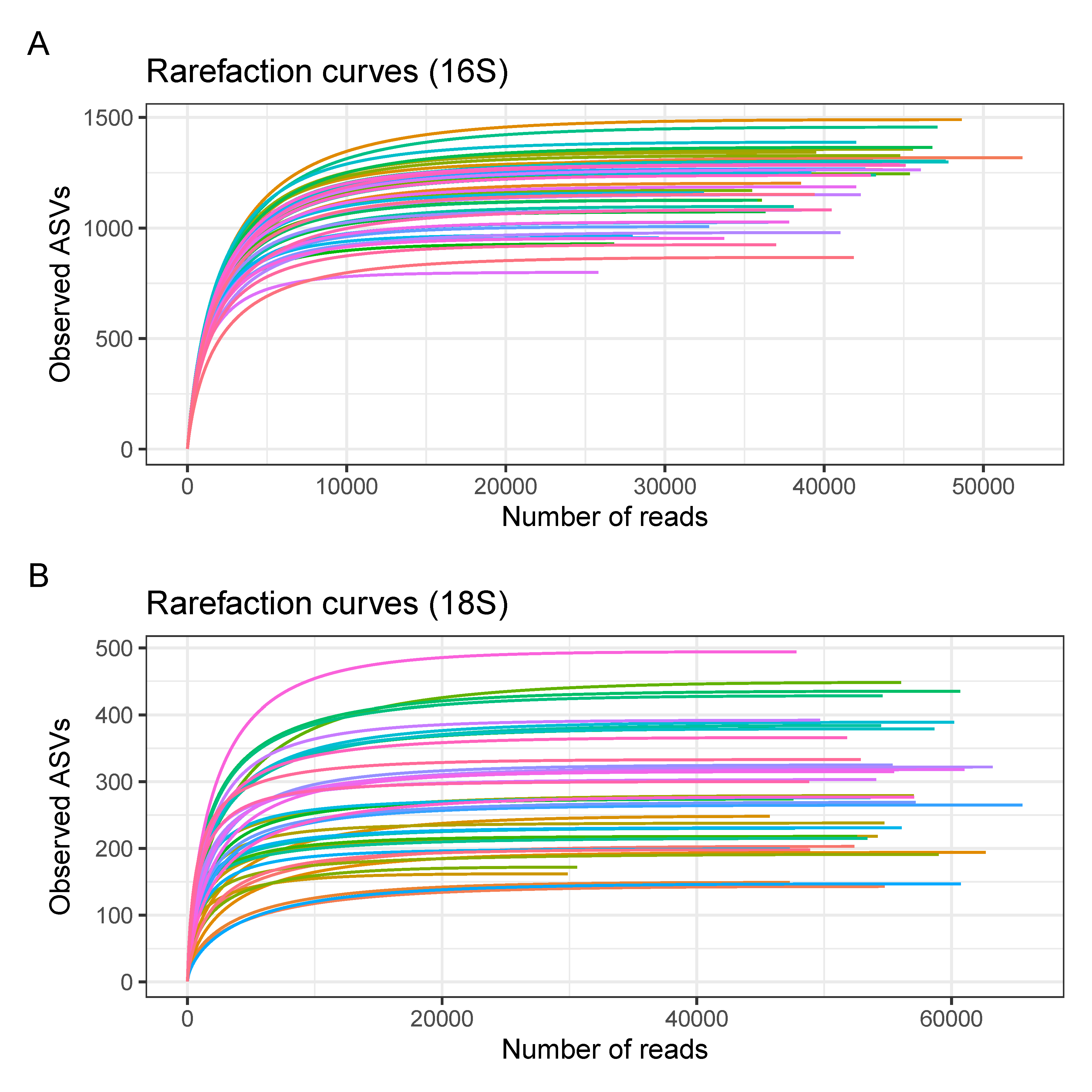

Supplement: Supplementary_material_ycag172 [file supplementary_material_ycag172.zip › Fig. S1rarefaction_curve.tiff]
